# Subjective well-being predicts health behavior in a population-based 9-years follow-up of working-aged Finns

**DOI:** 10.1016/j.pmedr.2021.101635

**Published:** 2021-11-14

**Authors:** Säde Stenlund, Heli Koivumaa-Honkanen, Lauri Sillanmäki, Hanna Lagström, Päivi Rautava, Sakari Suominen

**Affiliations:** a Department of Public Health, University of Turku, Kiinamyllynkatu 10, 20014 Turku, Finland; b Research Services, Turku University Hospital, Kiinamyllynkatu 4-8, 20521 Turku , Finland; c Institute of Clinical Medicine, Psychiatry, University of Eastern Finland, Yliopistonranta 1A, 70029 Kuopio, Finland; d Mental Health and Wellbeing Center, Kuopio University Hospital, Puijonlaaksontie 2, 70029 Kuopio, Finland; e Department of Public Health, University of Helsinki, Tukholmankatu 8 B, 00014 Helsinki, Finland; f Centre for Population Health Research, University of Turku and Turku University Hospital, Kiinamyllynkatu 10, 20014 Turku, Finland; g School of Health Sciences, University of Skövde, Högskolevägen 1, 54128 Skövde, Sweden

**Keywords:** Subjective well-being, Life satisfaction, Health behavior, Longitudinal study, Follow-up, AIC, Akaike information criterion, HBSS, health behavior sum score, LS, life satisfaction, SWB, subjective well-being

## Abstract

The cross-sectional association between measures of subjective well-being (SWB) and various health behaviors is well-established. In this 9-year (2003–2012) follow-up study, we explored how a composite indicator of SWB (range 4–20) with four items (interest, happiness, and ease in life, as well as perceived loneliness) predicts a composite health behavior measure (range 0–4) including dietary habits, physical activity, alcohol consumption, and smoking status. Study subjects (n = 10,855) originated from a population-based random sample of working-age Finns in the Health and Social Support study (HeSSup). According to linear regression analysis, better SWB predicted better health behavior sum score with a β = 0.019 (p < 0.001) with a maximum effect of 0.3 points after adjusting for age (p = 0.038), gender (p < 0.001), education (p = 0.55), baseline self-reported diseases (p = 0.020), baseline health behavior (β = 0.49, p < 0.001), and the interaction between SWB and education (p < 0.001). The results suggest that SWB has long-term positive effect on health behavior. Thus, interventions aiming at health behavioral changes could benefit from taking into account SWB and its improvement in the intervention.

## Highlights

• Subjective well-being predicts subsequent health behavior in a 9-years of follow-up.
• Neither direction of influence was stronger as compared to the other one.
• Enhancing subjective well-being could serve as an additional support for health behavior change.

## 1 Introduction

Subjective well-being (SWB) refers to the extent to which a person perceives that his or her life is going well (Diener et al., 2018). It comprises at least cognitive and both positive and negative affective components (Busseri and Sadava, 2011). Canonical measures of life satisfaction (LS) typically ask people to evaluate their lives. Thus, LS has been considered as an indicator of SWB as it represents the cognitive component of SWB, whereas positive and negative affect represent emotional components of SWB (Diener et al., 2018). Both LS and SWB have been related to subsequent health and mortality (Koivumaa-Honkanen et al., 2000, Rissanen et al., 2011, Rauma et al., 2014, Diener et al., 2017a). Health behavior has been outlined as an important pathway in these relationships (Chida and Steptoe, 2008, Diener et al., 2017b, Ong, 2010, Steptoe et al., 2009, Hoogwegt et al., 2013). It is suggested e.g. that people higher in SWB have energy to engage in healthier behaviors such as exercise (Hoogwegt et al., 2013) or to avoid unhealthy behaviors such as heavy alcohol use and smoking (Diener et al., 2018, Koivumaa-Honkanen et al., 2012). Thus, deeper understanding about these relationships is needed (Diener et al., 2017b).

The association of SWB with health behavior has been established mainly in large cross-sectional studies (Diener et al., 2017b, Grant et al., 2009, Kushlev et al., 2019, Schnohr et al., 2005, Strine et al., 2008). In long-term settings, only a few studies have explored the association of SWB with subsequent health behavior (Diener et al., 2017b). Low levels of LS – using the same measure as in the present study – has predicted adverse alcohol consumption in a large population based sample aged 18–64 in a 15-year follow-up (Koivumaa-Honkanen et al., 2012). In cardiovascular patients, positive affect predicted survival, but it was largely explained by baseline physical activity (Hoen et al., 2013, Hoogwegt et al., 2013). An increase in positive affect across a five-year period co-occurred with improvements in their physical activity, but not in smoking status (Sin et al., 2015). In shorter follow-ups, high LS has predicted reduction in smoking, whereas bad mood has predicted smoking relapse in a 4-week website survey (Haller, 2016). In an up to 26-week smoking cessation trial, both high positive affect and low negative affect prevented relapses. Thus, positive affect and anhedonia have been suggested as additional targets in these interventions (Leventhal et al., 2008).

There are various measures that can be related to SWB such as dispositional factors gratitude and optimism (Carver et al., 2010, Diener et al., 2017b). Gratitude has been linked with better health outcomes, but the results in respect to health behavior are more mixed (Boggiss et al., 2020). In a follow-up from 6 months up to 10 years, inactive but happy and optimistic men showed significantly greater increase in physical activity than unhappy men. This did not apply to women (Baruth et al., 2011). Psychological well-being (including control, autonomy, self-realization, and pleasure) was associated with attaining and maintaining high levels of physical activity over a period of 11 years in adults aged ≥ 50 (Kim et al., 2017). According to an earlier review, the effect of positive personal well-being on subsequent health behavior was for the most explained by baseline health behavior (Boehm et al., 2012).

A few studies have explored the bidirectional nature of SWB and health behavior. Longitudinally, high alcohol consumption seemed to predict life dissatisfaction somewhat more strongly than vice versa in a 15-year follow-up of adults (Koivumaa-Honkanen et al., 2012). Similarly, smoking was a stronger predictor of poor personal well-being in a 4-year follow-up of older adults than vice versa (Lappan et al., 2018). In both studies, regardless of the direction, these results were statistically significant. In respect to diet, fruit and vegetable consumption predicted LS, but not vice versa in a 2-year follow-up of adults aged 15–93 years (Mujcic and Oswald, 2016). In our previous study, a composite indicator of health behavior predicted LS in a 9-year follow-up of working-aged Finns (Stenlund et al., 2021). The opposite direction has not, yet, been explored. As far as we know, previous studies have not explored the relationship between SWB and multiple domains of health behavior in both directions with same study sample. Thus, the bidirectional nature of this association needed further research (Boehm and Kubzansky, 2012, Diener et al., 2017b).

The aim of the present study was to explore whether a composite measure of SWB (including items on the interest, happiness, and ease in life, as well as perceived loneliness) predicts subsequent health behavior measured with a composite score for diet, physical activity, alcohol consumption, and smoking status.

## 2 Methods

### 2.1 Participants

**Fig. 1:**
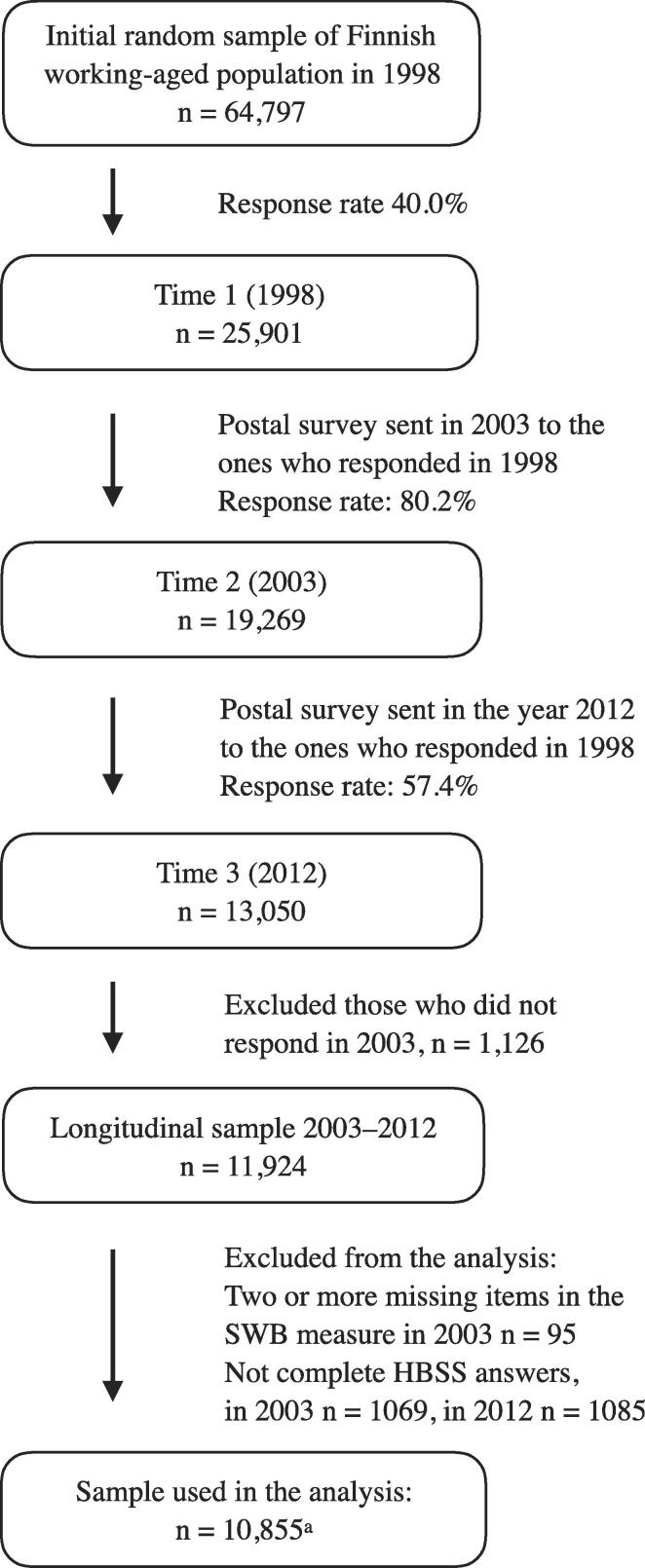
Inclusion and exclusion criteria for the present study population of the Health and Social Support (HeSSup) prospective population-based follow-up study. ^a^ Incomplete information of covariates or missing responses results in slightly different sample sizes in different analysis (n = 10,032–10,855). HBSS = Health behavior sum score SWB = Subjective well-being.

The prospective data on working-age Finns originated in a longitudinal postal survey on Health and Social Support (HeSSup) initiated in 1998 (Time 1). A random sample of the Finnish working-age population (n = 64,797) was drawn from the national population register to create four age groups: 20–24 years (group 1), 30–34 years (group 2), 40–44 years (group 3), and 50–54 years (group 4). Respondents gave their informed consent for prospective data collection**.** The baseline survey (Time 1) lacked measures of diet and SWB. Thus, in the present study, we utilize data collected from 2003 (Time 2) and 2012 (Time 3) of participants (n = 11,924) who had responded to all health behavior domains (n = 10,855, i.e. 91% of those who had responded both times). For details, see flow chart of the study (Fig. 1). Both surveys comprised identical items presented almost in an identical order. Additional details of the study population and attrition have been described elsewhere (Stenlund et al., 2021).

### 2.2 Measures

The four-item LS scale (range: 4–20) has been modified by Eric Allardt for Nordic welfare studies (Allardt, 1976). It has been used in several patient (Koivumaa-Honkanen et al., 2008, Koivumaa-Honkanen et al., 1996, Kuivasaari-Pirinen et al., 2014, Pakarinen et al., 2016, Salakari et al., 2016) and general population samples (Koivumaa-Honkanen et al., 2011, Koivumaa-Honkanen et al., 2000, Rauma et al., 2014, Rissanen et al., 2011). It has a strong long-term predictive ability for various long-term health outcomes (Koivumaa-Honkanen et al., 2000, Koivumaa-Honkanen et al., 2004, Rauma et al., 2014) and a strong concurrent association with and long-term predictive power to depression both in general (Koivumaa-Honkanen et al., 2004) and patients population samples (Koivumaa-Honkanen et al., 2008, Rissanen et al., 2011). Its four items include three assessments of life (interest, happiness, and ease in life) and perceived loneliness. The latter is beyond what is typical for a LS measure, but reflects social well-being, which is an aspect of SWB. Thus, we kept the measure to be consistent with previous studies but used it as an indicator of SWB as it is a broader concept. The four items were scored as follows: very interesting/ happy/ easy/ not at all lonely = 1; fairly interesting/ happy/ easy = 2; cannot say = 3; fairly boring/ unhappy/ hard/ lonely = 4; very boring/ unhappy/ hard/ lonely = 5. The lower score indicates higher SWB. The participants were categorized as previously into three groups: high SWB (4–6), the intermediate SWB group (7–11), and low SWB (12–20). The score was also used as a continuous variable in the linear regression analyses. If a response to a single item was missing (n = 54; 0.5%), it was replaced by the mean of the remaining items. Otherwise, the respondents with missing items on the score were excluded (n = 95, 0.9%).

*A health behavior sum score* (HBSS, range 0–4) is a count of the total number of protective health behaviors. It comprises four domains i.e. dietary habits, physical activity, alcohol consumption, and smoking status with risky behavior scoring 0 points and protective behavior scoring 1 point. *A dietary index* was formed to describe the adherence to dietary recommendations in line with Nordic Nutrition Recommendations (Becker et al., 2004). The following dietary items and frequencies or proportions were included in the short non-validated questionnaire: fat free milk (≥1/day); fish (≥1–2/week); dark bread, fresh fruits/berries or vegetables (each ≥ 2/day); pastries/sweets, sausages, red meat or chicken/turkey (each ≤ 1–2/week); alcohol consumption (<10 g women, 20 g men/day) (Lagström et al., 2019). Each of them provided one point if the criterion was fulfilled. Thus, the range of the overall score covered the interval 0 to 10 (best). The score was multiplied by 10 to create a percentage scale for the statistical analyses (range: 0–100). *Physical activity* was a measure based on the intensity and time spent on physical activity in leisure time or in commuting (hours in week). It was converted to Metabolic Equivalent Task (MET) with a MET value 2 corresponding to approximately 30 min of walking per day and being the cut-off point for physically active individuals (Kujala et al., 2002). *Smoking status* was dichotomized: current smokers vs. former smokers or never being a regular smoker. *Alcohol consumption* was converted to grams per week. The cut-off point for heavy drinkers was ≥ 140 g/week for women and ≥ 280 g/week for men according to concurrent Finnish guidelines (Alho et al., 2010).

Categories for the four domains of the HBSS were defined as follows:

Dietary patterns: sum score ≤ 50, HBSS = 0 vs. sum score ≥ 60, HBSS = 1.
Physical activity: inactive MET < 2, HBSS = 0 vs. active MET ≥ 2, HBSS = 1.
Smoking status: smokers, HBSS = 0 vs. former smokers or never being a regular smoker, HBSS = 1.
Alcohol consumption: heavy drinkers (women ≥ 140 g/week; men ≥ 280 g/week), HBSS = 0 vs. non-drinkers and moderate drinkers, HBSS = 1.

*Education and health* can affect both health behavior and SWB (Cowell, 2006, Dolan et al., 2008, Zanjani et al., 2006)). In the analyses, education at baseline was categorized into four groups: 1) no professional education; 2) vocational course/ school/ apprenticeship contract; 3) college school; 4) university degree/ university of applied sciences. Health status was measured by self-report from a pre-defined list of 35 diseases at baseline and categorized into the following three groups: 0, 1, ≥ 2 diseases. The 35 diseases in the survey can be found in the Appendix A. The severity of diseases was not measured in the survey and it can vary considerable within a condition. Thus, the burden caused by a specific disease cannot be determined. As multimorbidity has been shown to be an important health indicator (Kivimäki et al., 2017), and disease count is one of its main measures (Lee et al., 2021) we also found the grouping of participants according to the number of diseases as appropriate. Multimorbidity was a statistically significant covariate in the linear regression models of the present study. Previously, it has also shown a linear association with our SWB measure (Lukkala et al., 2016).

### 2.3 Statistical analysis

As the correlation between baseline SWB_2003_ and subsequent HBSS_2012_ appeared linear, multiple linear regression models were investigated. The Cronbach’s alpha was computed for SWB_2003_. Model 1 explored first the crude association between SWB_2003_ and HBSS_2012_. In model 2, age, gender, education, and major diseases at baseline were added as covariates. In the model 3, health behavior at baseline (HBSS_2003_) was added to the model 2. The interactions between SWB_2003_ and the covariates (age, gender, education, and major diseases) were tested as a differing effect of SWB_2003_ on health behavior might be observed between different covariate groups e.g. the effect of SWB might differ by sex. The SWB_2003_*education was the only significant interaction and was included to create model 4. Further, the association of SWB_2003_ and each subsequent health behaviors were analyzed separately according to models 1–3 above but using logistic regression models whereafter the Akaike information criterion (AIC) of the models adjusted for all covariates were compared. We did not include depression as a covariate in the models to avoid overadjusting. Data were analyzed with SAS software (version 9.4; SAS Institute Inc. Cary, NC, USA 2016).

## 3 Results

The average SWB_2003_ for the study population was 8.53 (SD 3.20). The intermediate SWB_2003_ group showed the highest proportion in the entire study population (56.8%) compared to the high SWB (25.1%) or to the low SWB groups (18.1%). After follow-up, having three favorable health behaviors was the most common (40.8%) followed with having all four (32.0%), two (21.1%), one (5.4%), or none (0.8%). Women and older people showed higher representation. Details of the baseline characteristics and outcome averages for subgroups are given in Table 1. Categorical SWB differed significantly (p < 0.001) by education and number of diseases but not by gender or age groups. The Cronbach’s alpha for SWB_2003_ was 0.76.

**Table 1:** Baseline characteristics (in 2003) and outcome (in 2012) by study characteristics. Results of the Finnish population-based HeSSup-study.

Variable	Level	Share of the study population^b^, % (n)	SWB_2003_^a^ mean (SD)	Low SWB score % (n)	Intermediate SWB score % (n)	High SWB score % (n)	HBSS_2003_ Mean (SD)	HBSS_2012_ mean (SD)	HBSS_2012_ = 0 % (n)	HBSS_201 2_ = 1 % (n)	HBSS_2012_ = 2 % (n)	HBSS_2012_ = 3 % (n)	HBSS_2012_ = 4 % (n)
Whole study population			8.53(3.20)	18.1(1,949)	56.8(6,110)	25.1(2,696)	2.87(0.90)	2.99(0.99)	0.8(90)	5.4(6 3 8)	21.1(2,496)	40.8(4,831)	32.0(3,798)

Age (2003)	25–29	21.3(2,529)	8.47(3.15)	19.0(4 2 7)	51.7(1,160)	29.3(6 5 6)	2.91(0.84)	3.04(0.85)	0.5(12)	4.0(1 0 1)	20.6(5 2 2)	42.1(1,064)	32.8(8 3 0)
	35–39	21.2(2,509)	8.58(3.28)	18.7(4 1 9)	54.5(1,223)	26.8(6 0 2)	2.82(0.89)	2.96(0.91)	0.9(22)	6.3(1 5 7)	19.9(5 0 0)	42.6(1,069)	30.3(7 6 1)
	45–49	26.4(3,131)	8.64(3.31)	18.8(5 3 8)	58.1(1,663)	23.2(6 6 4)	2.82(0.94)	2.94(0.94)	1.1(33)	6.8(2 1 4)	22.1(6 9 1)	38.2(1,196)	31.8(9 9 7)
	55–59	31.1(3,684)	8.45(3.06)	16.6(5 6 5)	60.7(2,064)	22.7(7 7 4)	2.91(0.91)	3.01(0.88)	0.6(23)	4.5(1 6 6)	21.3(7 8 3)	40.8(1,502)	32.8(1,210)

Gender	Male	36.7(4,345)	8.58(3.18)	17.6(6 8 4)	59.4(2,302)	23.0(8 9 3)	2.66(0.92)	2.82(0.92)	1.1(49)	7.4(3 2 0)	25.4(1,105)	42.0(1,823)	24.1(1,048)
	Female	63.3(7,508)	8.51(3.20)	18.4(1,265)	55.4(3,808)	26.9(1,803)	2.98(0.87)	3.09(0.87)	0.6(41)	4.2(3 1 8)	18.5(1,391)	40.1(3,008)	36.6(2,750)

Education (2003)	No professional education	12.0(1,298)	8.89(3.33)	21.5(2 7 7)	58.0(7 4 7)	20.6(2 6 5)	2.67(0.93)	2.81(0.92)	0.8(10)	7.2(93)	26.9(3 4 9)	40.2(5 2 2)	25.0(3 2 4)
	Vocational school	29.0(3,126)	8.76(3.30)	20.1(6 2 0)	58.1(1,797)	21.8(6 7 5)	2.71(0.92)	2.83(0.94)	1.02(32)	7.7(2 4 0)	24.9(7 7 9)	39.5(1,236)	26.8(8 3 9)
	College	39.0(4,204)	8.40(3.15)	17.1(7 1 2)	56.6(2,363)	26.4(1,100)	2.91(0.89)	3.03(0.89)	0.7(30)	4.7(1 9 6)	19.3(8 1 3)	41.3(1,734)	34.0(1,431)
	University	20.0(2,163)	8.24(3.01)	15.4(3 3 3)	54.5(1,176)	30.0(6 4 8)	3.13(0.81)	3.23(0.77)	0.3(6)	1.4(30)	14.8(3 2 0)	41.9(9 0 7)	41.6(9 0 0)

Diseases(2003)	0	18.0(1,935)	7.84(2.73)	11.8(2 2 8)	56.9(1,097)	31.2(6 0 2)	2.94(0.86)	3.06(0.86)	0.5(10)	4.0(77)	19.3(3 7 4)	41.1(7 9 6)	35.0(6 7 8)
	1	23.4(2,522)	8.12(2.94)	14.5(3 6 3)	56.0(1,399)	29.5(7 3 7)	2.89(0.88)	3.04(0.89)	0.8(19)	4.8(1 2 2)	18.5(4 6 7)	41.9(1,057)	34.0(8 5 7)
	2 or more	58.7(6,323)	8.91(3.37)	21.5(1,347)	57.1(3,586)	21.5(1,348)	2.84(0.92)	2.95(0.91)	0.8(50)	5.7(3 5 9)	22.3(1,410)	40.3(2,549)	30.9(1,955)

SWB_2003_^a^	Low SWB score 12–20	18.1(1,949)	5.38(0.68)	100	0	0	2.63(0.97)	2.77(0.98)	1.6(31)	9.1(1 7 7)	25.4(4 9 4)	38.3(7 4 6)	25.7(5 0 1)
	Intermediate SWB score 7–11	56.8(6,110)	8.14(1.35)	0	100	0	2.87(0.88)	3.00(0.88)	0.6(36)	4.8(2 9 0)	20.9(1,278)	41.8(2,551)	32.0(1,955)
	High SWB score 4–6	36.7(4,345)	14.14(2.09)	0	0	100	3.03(0.85)	3.12(0.84)	0.4(11)	3.3(90)	17.8(4 7 9)	40.4(1,089)	38.1(1027)

HBSS = Health behavior sum score i.e. number of protective health behaviors.

SWB = Subjective well-being.

a lower score indicating better subjective well-being.

b Subgroup sizes vary slightly due to missing responses.

SWB_2003_ predicted both subsequent health behavior domains as well as HBSS_2012_ in all the studied linear models. Adjusting for covariates age, gender, education, major diseases, and HBSS_2003_ progressively decreased the estimate for slope and improved the AIC. Adjusting for the only significant interaction slightly increased both the slope and AIC. For details see Table 2. In the final Model 4 (Table 3), with the interaction term SWB_2003_*education, the slope for SWB_2003_ was –0.019. Due to reversed LS score, it meant that every point towards better SWB (i.e. lower score; remind of range 4–20) improves HBSS_2012_ by 0.019 points with a maximum effect of 0.3 points (range 0–4). Increase of HBSS_2003_ with one point predicted 0.49 higher HBSS_2012_. Worse HBSS_2012_ was observed in the middle-aged compared to other age groups (p = 0.038), in men compared to women (p < 0.001), and in those having two or more diseases compared to the ones with one or no diseases (p = 0.020). The interaction term SWB_2003_*education was also statistically significant in which university or higher education had stronger effect on how SWB_2003_ predicts HBSS_2012_ (p < 0.001) compared to lower education. However, education by itself was not a significant covariate.

**Table 2:** Estimates of linear models in which a continuous subjective well-being measure in 2003 predict subsequent health behavior sum score in 2012.

Model	Estimate	Standard error	p-value	AIC
Model 1: Crude linear model: no covariates	–0.038	0.0027	<0.001	28,010
Model 2: Model 1 + Age, gender, education, diseases	–0.033	0.0027	<0.001	27,320
Model 3: Model 2 + HBSS_2003_	–0.014	0.0024	<0.001	23,100
Model 4: Model 3 + SWB_2003_*education	–0.019	0.0043	<0.001	23,110

Results of Finnish population-based HeSSup-study.

SWB_2003_ = Subjective well-being at baseline in 2003, lower scores indicating better SWB.

HBSS_2003_ = Health behavior sum score i.e. number of protective health behaviors at baseline in 2003.

**Table 3:** Estimates of the multi-variable linear regression model 4 in which a subjective well-being measure in 2003 predicts health behavior sum score in 2012.

	Level	Estimate	Standard error	p-value
Intercept		1.75	0.051	<0.001
SWB_2003_		–0.019	0.0043	<0.001
HBSS_2003_		0.49	0.0088	<0.001

Age (1998)	20–24	–0.031	0.023	0.017
	30–34	–0.041	0.022	0.066
	40–44	–0.058	0.020	0.005
	50–54	Reference		

Gender	Male	–0.10	0.016	<0.001
	Female	Reference		

Education (1998)	No professional education	4.8*10^−6^	0.076	1.0
	Vocational school	Reference		
	College	0.047	0.053	0.37
	University or higher	–0.036	0.064	0.58

Diseases (2003)	0	0.051	0.021	0.018
	1	0.041	0.019	0.032
	2 or more	Reference		

SWB_2003_*education	No professional education	–8.6*10^-4^	0.0079	0.91
	Vocational school	Reference		
	College	0.0030	0.0058	0.60
	University or higher	0.024	0.0071	< 0.001

Results of Finnish population-based HeSSup-study.

SWB_2003_ = Subjective well-being at baseline in 2003, lower scores indicating better subjective well-being.

HBSS_2003_ = Health behavior sum score score i.e. number of protective health behaviors at baseline in 2003.

Diseases (2003) = Number of the diseases participants reported at baseline in 2003. List of diseases is found in the Appendix A.

In logistic regression models, SWB_2003_ predicted also individual health behavior domains nine years later when adjusted for age, gender, education, major diseases, and the baseline level of the specific health behavior (all p < 0.001). The AIC were lowest for prediction of alcohol consumption (AIC = 3,242) and smoking status (AIC = 4,253) compared to the association to subsequent physical activity (AIC = 10,971) and dietary habits (AIC = 12,552). Details of the models for individual health behaviors can be found in the Appendix B.

## 4 Discussion

### 4.1 Main findings

In a nine-year follow-up of 10,000 randomly sampled working-aged Finns, a composite measure of subjective well-being (SWB) – including happiness, interest, and ease in life, as well as perceived loneliness – predicted a composite indicator of four main health behaviors in respect to their public health importance. This was shown also when age, gender, education, number of diseases, health behavior at baseline as well as interaction between education and SWB were controlled. Even if the strongest covariate was the baseline health behavior and it halved the estimate, it did not remove the statistical significance in the longitudinal association of SWB and health behavior.

Previously, we have reported the opposite direction of prediction between health behavior and SWB with the same measure, data set, and nine-year follow-up time (Stenlund et al., 2021). It showed that baseline health behavior (HBSS_2003_) predicted independently SWB (in 2012) after adjusting for the same covariates – only SWB_2003_ being replaced by HBSS_2003_ and without interactions. In both directions, the ratios between the estimates of prediction and standard deviation or range of scale of the independent variable were equal to one significant figure. Thus, neither direction is clearly stronger, but there are other differences. Gender and the interaction term between baseline SWB and level of education are significant covariates in the present study but not in the opposite direction of influence. When individual health behaviors were explored, the strongest association (lowest AIC) was observed when non-smoking predicted SWB in our previous study and when SWB predicted alcohol consumption and smoking in the present study.

It is always important to acknowledge what is assessed in different SWB or LS (life satisfaction) measures. In the present study, SWB measured with three self-perceived assessments of life (happiness/ interest/ ease in life) and one of loneliness, enhanced health behavior. Perceived loneliness is not typically conceptualized as a part of LS measures, but it reflects social well-being i.e. aspect of SWB. Furthermore, this SWB measure resulted in an acceptable level of Cronbach’s alpha indicating it being a consistent measure. Previous studies have shown a longitudinal relationship between diverse measures and components of SWB and health behavior where several pathways can be suggested. One pathway could be through SWB and increased self-efficacy to health behavior changes, which require personal effort. A positive affect intervention (with three RCTs) increased both self-efficacy and promoted success in behavioral change by buffering stress in patients with chronic cardiopulmonary disease (Charlson et al., 2014). Thus, interventions aiming to changes in health behavior could benefit from taking into account also SWB and its determinants (Charlson et al., 2014, Diener et al., 2017b, Kushlev et al., 2020, Lyubomirsky and Layous, 2013). In the present study, the effect of SWB_2003_ was stronger for those with higher education. Better health literacy and better knowledge about health behavior can also increase the effect of SWB on health behavior and result in beneficial health behavior changes. Similar results have also been reported for diet and physical activity in patients with cardiovascular diseases (Aaby et al., 2017) and diabetes (Friis et al., 2016).

To the best of our knowledge, the present study is the first to explore the relationship between a composite measure of SWB and a composite health behavior measure with an extensive follow-up enabling a bidirectional comparison of this relationship when combined with our previous study exploring the opposite direction (Stenlund et al., 2021). In previous bidirectional studies with only single addiction-based health behavior, good health behavior has predicted better SWB somewhat more than vice versa in respect to alcohol use in a 15-year follow-up (Koivumaa-Honkanen et al., 2012) and in respect to smoking status in a four-year follow-up (Lappan et al., 2018), whereas fruit and vegetable consumption predicted better LS but not vice versa in a two-year follow-up (Mujcic and Oswald, 2016). In shorter follow-up times and without bidirectional setting, better outcomes have been observed in affect predicting smoking cessation (Leventhal et al., 2008), mood predicting smoking relapse, and SWB predicting reduction in smoking (Haller, 2016).

During a long follow-up, there can be changes in many aspects of life, which in turn can influence both SWB and health behavior. Thus, it is important to acknowledge that a statistically significant association between SWB at baseline and subsequent health behavior was observed after nine years. The role of baseline health behavior was underlined and it accounted for half of the variation of subsequent health behavior. Further, these results were obtained with composite measures of both SWB and health behavior. Thus, the results do not inform of the effect of a specific change, but rather describe more generally how the two concepts relate to each other. This kind of information can enable policy makers, health care, and society to confront these health adversities and to create strategies to promote both SWB and the four main domains of health behaviors. Even though the effect size is fairly modest it is still significant in describing trends in the population*.* The maximum effect of SWB predicting health behavior corresponds also to almost a third of the standard deviation of the mean of SWB. The effect equals also to the difference in SWB between the highly educated and those without any professional education.

### 4.2 Implications

The burden of chronic diseases and increasing expenditures in medical care challenges health care systems around the world (OECD, 2019). SWB could serve as a supportive strategy for achieving favorable health behavior. As multiple health behaviors were included as a composite measure the results inform policy makers that better levels of SWB result in better health behaviors. Change in any of the four main domains of health behavior is beneficial and can reduce chronic conditions. However, the effect of SWB seems to be strongest for smoking status and alcohol consumption. Nevertheless, targeting also SWB when aiming at health behavior changes could give additional benefits and, hence, result in overall better health for individuals.

### 4.3 Strengths and limitations

The study has several strengths. Our data was large, follow-up was nine years, and enabled the exploration of the bidirectional relationship between SWB and multiple health behavior when combined with our previous study (Stenlund et al., 2021). The large population-based sample and a consistent survey procedure can provide solid results. To the best of our knowledge, this is the first study to include a composite measure for both SWB and health behavior. The four studied health behavior domains all represented the main public health concerns. These composite measures cover health behavior and SWB more broadly rather than when focusing only on single aspects of health behavior and SWB. However, we made also separate analyses for each type of healthy behavior to complete the picture. Together with our previous study, policy makers and health care can be informed about general trends in a population. The attrition in the follow-up did not result in such changes that could decrease the statistical significance of the found associations or the representativeness of the study subjects to general population (Stenlund et al., 2021). Studying individuals with low and high SWB could give tools for health promotion also in mental health.

A study limitation was that only two time points could be used since the baseline survey (Time 1) lacked measures of diet and SWB. Multiple follow-up points would have enabled a more detailed analysis. Since data collection, society and health behaviors have changed. E.g. smartphones and social media have become an essential part of everyday life. These changes and their effects (such as increased screen time and sedentary life) need to be explored to understand what kind of new approaches to health promotion are required. SWB research employs a large variety of measures from single item questions to multiple item measures, but none has become a golden standard (Pavot, 2018). Thus, acknowledging what is assessed in different SWB measures is crucial for understanding and comparisons.

### 4.4 Further research

More research is needed to compare the effect of different concepts and components of SWB measures. Further, exploring a wider variety of SWB indicators such as optimism, measures of coping, sense of coherence, and resilience deepens the knowledge between SWB and health behavior. Lastly, intervention studies focusing on improving SWB and health behavior are needed due to their great importance in health promotion, health, and thus also in policymaking.

## 5 Conclusion

Subjective well-being predicted health behavior after nine years among working-age Finns. Thus, SWB has long-term positive effect on health behavior. Interventions aiming at health behavioral changes could benefit from taking into account also SWB and its improvement.

## 6 Ethics

The concurrent joint Ethical Committee of the University of Turku and the Turku University Central Hospital approved the Health and Social Support study. This study was carried out according to the Declaration of Helsinki. Participants signed a written consent agreeing to a prospective follow-up.

## 7 Data Availability

The study data contains variables of personal and sensitive nature and hence, due to the present legislation cannot be made openly accessible inside or outside Finland.

## Funding

This work was supported by personal grants by the Signe and Ane Gyllenberg foundation (SSt:5175 and HKH: 5525) [2020]; and the Waldemar von Frenckel foundation [2020] (SSt) and The Päivikki ja Sakari Sohlberg Foundation (HKH:2020) [2020]. The funding sources did not participate in the design or conduct of the study; collection, management, analysis, or interpretation of the data; or preparation, review, or approval of the manuscript.

### CRediT authorship contribution statement

**Säde Stenlund:** Conceptualization, Methodology, Investigation, Writing – original draft, Writing – review & editing, Visualization, Project administration, Funding acquisition. **Heli Koivumaa-Honkanen:** Conceptualization, Methodology, Investigation, Resources, Writing – review & editing, Supervision. **Lauri Sillanmäki:** Methodology, Formal analysis, Data curation, Writing – review & editing. **Hanna Lagström:** Conceptualization, Writing – review & editing. **Päivi Rautava:** Investigation, Resources, Writing – review & editing, Supervision. **Sakari Suominen:** Conceptualization, Methodology, Investigation, Resources, Writing – review & editing, Supervision.

## Declaration of Competing Interest

The authors declare that they have no known competing financial interests or personal relationships that could have appeared to influence the work reported in this paper.
