# Supplementary material for: Subjective well-being predicts health behavior in a population-based 9-years follow-up of working-aged Finns
Source: Prev Med Rep. 2021 Nov 14;24:101635. doi: 10.1016/j.pmedr.2021.101635 (PMC8684019; doi:10.1016/j.pmedr.2021.101635)
Supplement: Supplementary Data 1 [file mmc1.pdf]

Appendix A: List of Diseases

Survey question on diseases in Health and Social Support study (originally in Finnish or Swedish):

Has a doctor ever said, that you have or had had any of the following conditions (yes/no):

Long term bronchitis or bronchiectasis

Lung asthma

Allergic rhinitis e.g. hay fever

High blood pressure

Hypertension

High cholesterol

Diabetes

Myocardial infarction or coronary thrombosis

Angina pectoris, i.e. chest pain caused by coronary artery disease

Atrial fibrillation or atrial flutter

Stroke

Other cerebrovascular accident

Peptic ulcer

Celiac disease

Liver disease

Kidney disease

Rheumatoid arthritis

Arthrosis

Sciatica

Fibromyalgia

Cataract or glaucoma

Migraine

Epilepsy

Brain injury

Meningitis or encephalitis

Other cerebral disease of neurological disease

Depression

Panic disorder

Eating disorder

Other mental disorder

Malignant tumor

Other chronic or severe disease, which?
