# Supplementary material for: Subjective well-being predicts health behavior in a population-based 9-years follow-up of working-aged Finns
Source: Prev Med Rep. 2021 Nov 14;24:101635. doi: 10.1016/j.pmedr.2021.101635 (PMC8684019; doi:10.1016/j.pmedr.2021.101635)
Supplement: Supplementary Data 2 [file mmc2.pdf]

Appendix B: Models on Individual Health Behaviors

Table B1 Non-standardized estimates for the logistic regression model in which baseline subjective well-being predicts subsequent dichotomized dietary habits. Observed statistical significance level of the covariates. The Finnish population-based Health and Social Support Study.

Model	AIC	Intercept (p-value)	SWB_{2003} [a] (p-value)	Dietary habits 2003 (p-value)	Age	Gender	Education	Diseases
Model 1: Crude logistic model where SWB_{2003}[a] predicts dietary habits in 2012, no covariates	14,817	-0.26 (< 0.001)	0.045 (< 0.001)	-	-	-	-	-
Model 2: Model 1 + gender, age, education, diseases as covariates	14,313	0.66 (< 0.001)	0.041 (< 0.001)	-	< 0.001	< 0.001	< 0.001	0.64
Model 3: Model 2 + dietary habits in 2003 as a covariate	12,552	1.01 (< 0.001)	0.025 (0.003)	-1.32 (< 0.001)	< 0.001	< 0.001	< 0.001	0.27

[a] SWB_{2003}= subjective well-being in 2003; lower score indicates better subjective well-being.

Table B2 Non-standardized estimates for the logistic regression model in which baseline subjective well-being predicts subsequent dichotomized physical activity. Observed statistical significance level of the covariates. The Finnish population-based Health and Social Support Study.

Model	AIC	Intercept (p-value)	SWB_{2003} [a] (p-value)	Physical activity 2003 (p-value)	Age	Gender	Education	Diseases
Model 1: Crude logistic model where SWB_{2003}[a] predicts physical activity in 2012, no covariates	12,853	-1.31 (< 0.001)	0.047 (< 0.001)	-	-	-	-	-
Model 2: Model 1 + gender, age, education, diseases as covariates	12,553	-1.34 (< 0.001)	0.041 (< 0.001)	-	< 0.001	0.006	< 0.001	0.008
Model 3: Model 2 + physical activity in 2003 as a covariate	10,971	-0.27 (0.029)	0.030 (< 0.001)	-1.42 (< 0.001)	< 0.001	0.15	< 0.001	0.011

[a] SWB_{2003}= subjective well-being in 2003; lower score indicates better subjective well-being.

Table B3 Non-standardized estimates for the logistic regression model in which baseline subjective well-being predicts subsequent dichotomized smoking status. Observed statistical significance level of the covariates. The Finnish population-based Health and Social Support Study.

Model	AIC	Intercept (p-value)	SWB_{2003} [a] (p-value)	Smoking status 2003 (p-value)	Age	Gender	Education	Diseases
Model 1: Crude logistic model where SWB_{2003}[a] predicts smoking status in 2012, no covariates	8,526	-2.65 (< 0.001)	0.092 (< 0.001)	-	-	-	-	-
Model 2: Model 1 + gender, age, education, diseases as covariates	8,116	-1.86 (< 0.001)	0.08 (< 0.001)	-	< 0.001	< 0.001	< 0.001	0.032
Model 3: Model 2 + smoking status in 2003 as a covariate	4,253	0.41 (0.035)	0.043 (0.0003)	-4.20 (< 0.001)	< 0.001	0.82	< 0.001	0.70

[a] SWB_{2003}= subjective well-being in 2003; lower score indicates better subjective well-being.

Table B4 Non-standardized estimates for the logistic regression model in which baseline subjective well-being predicts subsequent dichotomized alcohol consumption. Observed statistical significance level of the covariates. The Finnish population-based Health and Social Support Study.

Model	AIC	Intercept (p-value)	SWB_{2003} [a] (p-value)	Alcohol consumption 2003 (p-value)	Age	Gender	Education	Diseases
Model 1: Crude logistic model where SWB_{2003}[a] predicts alcohol consumption in 2012, no covariates	4,480	-3.64 (< 0.001)	0.087 (< 0.001)	-	-	-	-	-
Model 2: Model 1 + gender, age, education, diseases as covariates	4,345	-3.83 (< 0.001)	0.080 (< 0.001)	-	< 0.001	< 0.001	0.83	0.061
Model 3: Model 2 + alcohol consumption in 2003 as a covariate	3,242	-0.98 (< 0.001)	0.047 (< 0.001)	-3.27 (< 0.001)	< 0.001	< 0.001	0.63	0.066

[a] SWB_{2003}= subjective well-being in 2003; lower score indicates better subjective well-being.
